# Spiral SAR Imaging with Fast Factorized Back-Projection: A Phase Error Analysis

**DOI:** 10.3390/s21155099

**Published:** 2021-07-28

**Authors:** Juliana A. Góes, Valquiria Castro, Leonardo Sant’Anna Bins, Hugo E. Hernandez-Figueroa

**Affiliations:** 1 School of Electrical and Computer Engineering, University of Campinas—UNICAMP, Campinas 13083-852, Brazil; v162627@g.unicamp.br (V.C.); hugo@unicamp.br (H.E.H.-F.); 2 National Institute for Space Research—INPE, São José dos Campos 12227-010, Brazil; leonardo.bins@inpe.br

**Keywords:** synthetic aperture radar (SAR), fast factorized back-projection (FFBP), 3D imaging, drone

## Abstract

This paper presents a fast factorized back-projection (FFBP) algorithm that can satisfactorily process real P-band synthetic aperture radar (SAR) data collected from a spiral flight pattern performed by a drone-borne SAR system. Choosing the best setup when processing SAR data with an FFBP algorithm is not so straightforward, so predicting how this choice will affect the quality of the output image is valuable information. This paper provides a statistical phase error analysis to validate the hypothesis that the phase error standard deviation can be predicted by geometric parameters specified at the start of processing. In particular, for a phase error standard deviation of ~12°, the FFBP is up to 21 times faster than the direct back-projection algorithm for 3D images and up to 13 times faster for 2D images.

## 1. Introduction

In synthetic aperture radar (SAR) imaging, circular flight path surveys produce 2D images with very high resolution as data are collected over 360° around the imaged area. Circular SAR can also provide 3D scattering information, but the 3D images are deformed by strong cone-shaped sidelobes [1,2,3]. Multicircular SAR, or holographic SAR tomography (HoloSAR), creates another synthetic aperture in elevation that mitigates these undesirable sidelobes, thus providing complete 3D data reconstruction with very high resolution [4,5,6,7,8,9]. HoloSAR geometry acquisition consists of multiple circular flight paths at different fixed heights. The sparse nature of the elevation aperture in HoloSAR poses some difficulties for a system working in the THz band [10]. These issues are overcome with a cylindrical spiral flight pattern with constant vertical speed.

SAR image processing requires efficient algorithms in terms of both accuracy and processing time. Frequency-domain algorithms are fast, but they perform better when the flight path is linear and free of motion errors. The time-domain back-projection (BP) algorithm can process SAR data for any flight path with high focusing quality but with high computational costs. Fast factorized back-projection (FFBP) algorithms can significantly reduce the computational time while still maintaining the accuracy of the BP algorithm. However, the increase in the level of sophistication makes it difficult to formulate an FFBP algorithm for arbitrary trajectories. As a result, many FFBP algorithms either assume a linear flight path to simplify calculations [11,12,13,14,15,16] or are tailored for circular flight paths [2,17,18,19]. 

In [20], the authors proposed an FFBP algorithm that describes subapertures through a data mapping approach that does not depend on the flight path geometry, even though the algorithm assumes that the radar constantly illuminates the imaged area or volume. Moreover, the algorithm operates in cartesian coordinates and employs a flexible tree structure that can handle both 2D and 3D data.

For the HoloSAR presented by Ponce et al. [4], different image layers were processed with a 2D FFBP that is customized for circular trajectories [2]. Ponce et al. did not pursue 3D focusing with their FFBP due to practical reasons [4]. Other HoloSAR solutions have used the direct BP algorithm [6,8], sparse reconstruction models [4,5,7], adaptive imaging [6,9], or a combination of them. Apart from the choice in the algorithm, there are two common approaches:

- Process each circular flight independently and merge the outputs [4,6];
- Make radial slices of the cylindrical synthetic aperture, process them separately, and then combine the results [5,7,8,9].

For the spiral SAR presented in [10], the whole trajectory was processed with the direct BP algorithm. In [20], the initial version of our FFBP algorithm successfully processed simulated SAR data of a spiral trajectory. To the best of our knowledge, it was the first full 3D FFBP algorithm capable of processing nonlinear SAR data.

Although the preliminary version of the FFBP algorithm [20] is fully functional, it has proven inefficient when operating with real SAR data, both in processing time and memory consumption. Therefore, this paper presents a more consolidated version of the FFBP algorithm [21] that employs vectorized variables and parallel processing to mitigate these issues. Vectorization is essential for increasing efficiency, while parallel computing further decreases processing time and reduces memory consumption.

Processing SAR data with an FFBP algorithm is not as straightforward as with a BP algorithm because some FFBP input parameters can affect the quality of the output image. Thankfully, Ulander et al. [12] provided an error analysis that yielded a method to limit the phase error by controlling the processing setup. 

This paper proposes a statistical phase error analysis inspired by [12] but with a key difference. Because the FFBP algorithm presented here works well with curved flight paths, the proposed analysis does not consider that deviations from a linear flight path will deteriorate the phase error.

The purpose was to test the hypothesis that geometric parameters at the beginning of processing can predict the phase error standard deviation of the output image. The data set for testing this hypothesis comprised processing results for a spiral flight path performed by a multiband drone-borne SAR system [22,23]. The collected P-band SAR data were processed with the BP and FFBP algorithms to produce 2D and 3D images. Different parameters were chosen for the FFBP to alter the response in phase error and processing time. 

The other sections of the paper are structured as follows. Section 2 presents the FFBP algorithm, the phase error hypothesis, and the case study. Section 3 evaluates several 2D and 3D SAR images regarding the phase error versus the signal-to-noise ratio (SNR), geometric parameters, and processing time. Finally, discussion of the results is presented in Section 4 and the conclusion in Section 5.

## 2. Materials and Methods

### 2.1. Fast Factorized Back-Projection Algorithm

**Figure 1:**
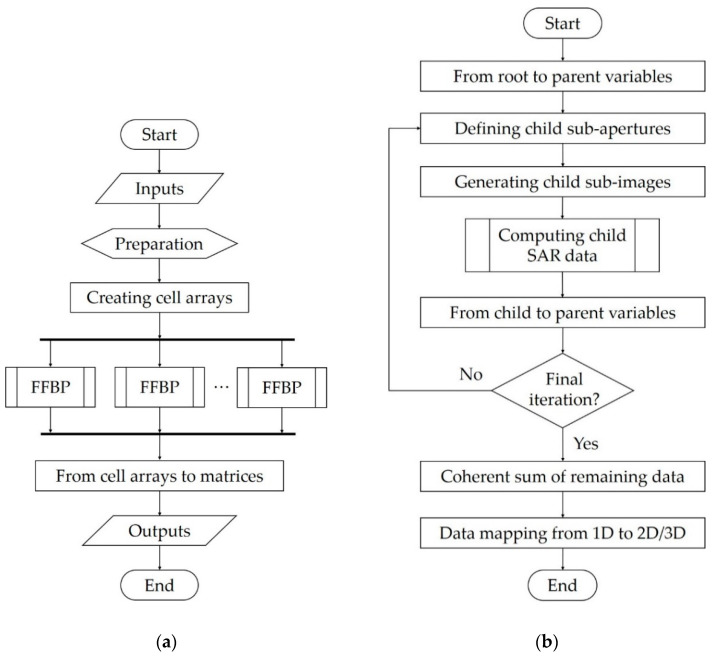
Flowchart of the algorithm: (**a**) parallel processing and (**b**) fast factorized back-projection (FFBP), an iterative process.

The BP algorithm integrates the information from all SAR positions for each pixel in the image in one go. If there are N SAR positions and the output image has $N^{2}$ pixels, the number of operations is $O\left(N^{3}\right)$. Fortunately, FFBP can reduce computational cost to $O\left(N^{2}\log N\right)$ using a divide-and-conquer strategy, which is at the core of many FFBP algorithms. Before processing starts, each SAR pulse covers a large area. At each iteration, subapertures are merged as if building increasingly larger antenna arrays with more focused beam patterns to cover progressively smaller subimages. Figure 1b shows the steps in this iterative process.

The FFBP algorithm is parallelizable, which means that the computation can be distributed among different processing units that work simultaneously. This is accomplished by dividing the imaged volume into blocks to be processed independently of one another. The data are managed by creating a cell array for each output matrix, i.e., processed SAR data and voxel coordinates. All cell arrays have the same number of elements, and each cell index is associated with an image block. When an image block is processed, its results are stored in the corresponding cells. Then, after processing all image blocks, each cell array is converted into a matrix that combines data for the whole output image. This process is illustrated in Figure 1a.

The next sections use the following terminology (see Figure 1):

- Root variables: either inputs to the algorithm or defined in the preparation step;
- Child variables: calculated within each FFBP iteration and then become parent variables at the end of the iteration;
- Parent variables: inputs to the current iteration.

The proposed algorithm is also vectorized, so matrix indices are written within parentheses to distinguish them from other types of indices. In addition, variables representing positions in the (*x*, *y*, *z*) space are written in bold letters.

#### 2.1.1. Defining Child Subapertures

The method for defining child subapertures was first proposed in [20]. It takes a data mapping approach and does not depend on the flight pattern. Let $r_{0}$ be the set of radar positions at the root node, let L be number of parent subapertures that are combined to form a child subaperture at each iteration, and let $r_{n}$ be the set of the phase centers of all child subapertures at the n^th^ node.

**Case** **1.** *When*L*is odd,*$r_{n}$*is always a subset of*$r_{0}$.

**Case** **2.** *When*L*is even, each point in*$r_{n}$*falls halfway between two consecutive points in*$r_{0}$.

**Figure 2:**
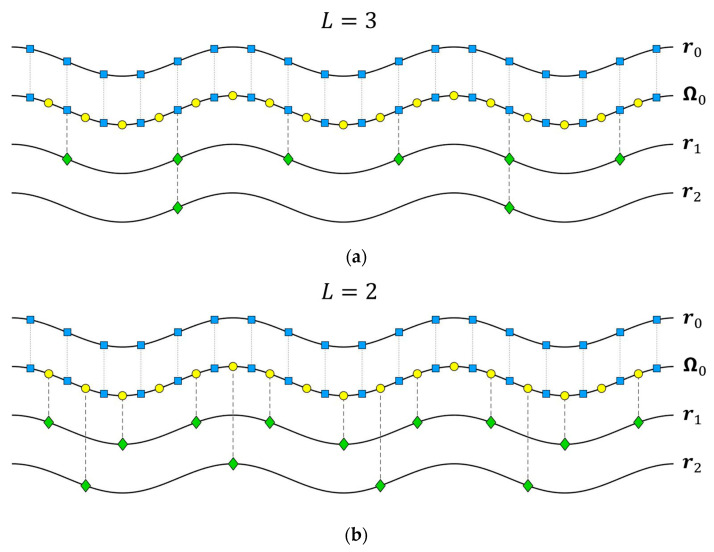
Definition of child subapertures for (**a**) L=3 and (**b**) L=2. The blue squares, yellow circles, and green diamonds represent the radar root positions, the midpoints between them, and the child subapertures phase centers, respectively. Reprinted with permission from ref. [20]. Copyright 2020 IEEE.

Cases 1 and 2 are depicted in Figure 2a,b, respectively. Blue squares represent the actual radar root positions, yellow circles represent the midpoints between them, and green diamonds represent the subaperture phase centers.

Now, let $\Omega_{0}$ be defined as

(1) Ω0(i)=r0(i2),

where $i=0,\;1,\;\ldots ,\;2\left(K_{0}-1\right)$, with $K_{0}$ being the number of radar root positions. Then, $\Omega_{0}$ is the union between $r_{0}$ and the set of midpoints between two consecutive radar root positions (see Figure 2).

**General** **Case.** *For any value of*L*,*$r_{n}$*is always a subset of*$\Omega_{0}$.

For the general case, $r_{n}$ is determined by [20]

(2) $$r_{n}\left(k\right)=\Omega_{0}\left(\left(2k+1\right)L^{n}-1\right),$$

where $k=0,\;1,\;\ldots ,\;K_{n}-1$, with $K_{n}=K_{0}/L^{n}$ being the number of child subapertures at the n^th^ node. Note that for all k, if L is odd, then the argument on the right will always be even, and vice versa.

#### 2.1.2. Generating Child Subimages

Child subimages are generated using a flexible space-filling tree structure called the modified Morton curve [20]. It arranges multidimensional data into 1D following a Z pattern, much like the original Morton order curve [24,25]. The modification, however, allows for different partition schemes beyond dividing by two in each direction in every recursion.

**Figure 3:**
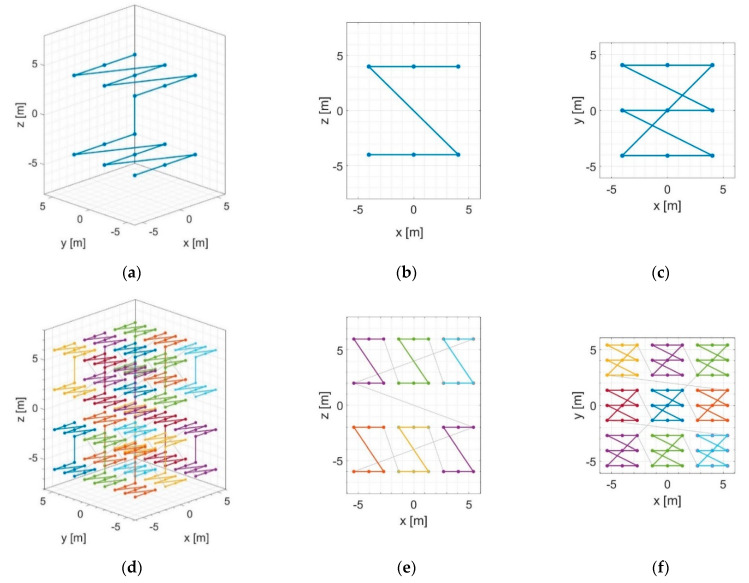
The modified Morton order curve with a (3 × 3 × 2) partition: (**a**) perspective, (**b**) front, and (**c**) top views for the first recursion; (**d**) perspective, (**e**) front, and (**f**) top views for the second recursion. Reprinted with permission from ref. [20]. Copyright 2020 IEEE.

The partition scheme for all iterations is defined in the preparation step (Figure 1a). It consists of a matrix whose columns contain the number of partitions in the *x*, *y*, and *z* dimensions ($D_{x},$ $D_{y}$, and $D_{z}$); the number of lines equals the number of iterations. These quantities are obtained from the output image dimensions and resolution, the initial subdivision into image blocks, and the number of combining subapertures L. Figure 3 shows the modified Morton order curve with a (3 × 3 × 2) partition on the first and second recursions. When working with 2D data, i.e., images with zero thickness, the partition scheme sets $D_{z}=1$ for all iterations.

After retrieving the partition scheme for the current iteration, the algorithm finds all possible values of *x*, *y*, and *z* coordinates for the center of the child subimages in a local coordinate system with the parent subimage center $h_{n-1}(p)$ at the origin. Next, the possible values of *x*, *y*, and *z* are arranged in a pattern similar to a truth table in digital systems theory to construct a Z-shaped curve of coordinates $\tilde{x}$, $\tilde{y}$, and $\tilde{z}$ (see Table 1). Then, the position of each child subimage center $h_{n}\left(c\right)$ is given by

(3) $$h_{n}\left(c\right)=\left[\begin{array}{ccc} \tilde{x}\left(d\right) & \tilde{y}\left(d\right) & \tilde{z}\left(d\right) \end{array}\right]+h_{n-1}\left(p\right),$$

(4) $$c=pD_{n}+d,$$

where $d=0,\;1,\;\ldots ,\;D_{n}-1$, $D_{n}=D_{x}D_{y}D_{z}$ is the number of children generated by each parent, p refers to a parent subimage, and c indicates a child subimage.

The positions $h_{n-1}\left(p\right)$ and $h_{n}\left(c\right)$ do not contain information about the terrain topography. Thus, the terrain height $H_{DEM}$ needs to be interpolated from a digital elevation model (DEM). Finally, the actual position of the child subimage ${\tilde{h}}_{n,c}$ is

(5) $${\tilde{h}}_{n,c}=h_{n}\left(c\right)+[0\;0\;H_{DEM}(h_{n}\left(c\right))].$$

To convert the serial index c into subscripts of a 3D matrix (u,v,w), recurrent sequences are necessary. These sequences are also built in a parent–child dynamic to allow for flexible partition schemes. Let $q_{x}{}_{n}$, $q_{y}{}_{n}$, and $q_{z}{}_{n}$ be the recurrent sequences of the n^th^ iteration, then

(6a) $$q_{x}{}_{0}\left(0\right)=q_{y}{}_{0}\left(0\right)=q_{z}{}_{0}\left(0\right)=0,$$

(6b) $$q_{x}{}_{n}\left(uD_{x}+d_{x}\right)=D_{n}q_{x}{}_{n-1}\left(u\right)+d_{x},$$

(6c) $$q_{y}{}_{n}\left(vD_{y}+d_{y}\right)=D_{n}q_{y}{}_{n-1}\left(v\right)+d_{y}D_{x},$$

(6d) $$q_{z}{}_{n}\left(wD_{z}+d_{z}\right)=D_{n}q_{z}{}_{n-1}\left(w\right)+d_{z}D_{x}D_{y},$$

where $d_{x}=0,\;1,\;\ldots ,\;D_{x}-1$, and the same for $d_{y}$ and $d_{z}$. Therefore, the mapping c→(u,v,w) from the modified Morton order curve into a 3D matrix can be carried out with the following relationship:

(7) $$c=q_{x}{}_{n}\left(u\right)+q_{y}{}_{n}\left(v\right)+q_{z}{}_{n}\left(v\right).$$

**Figure 4:**
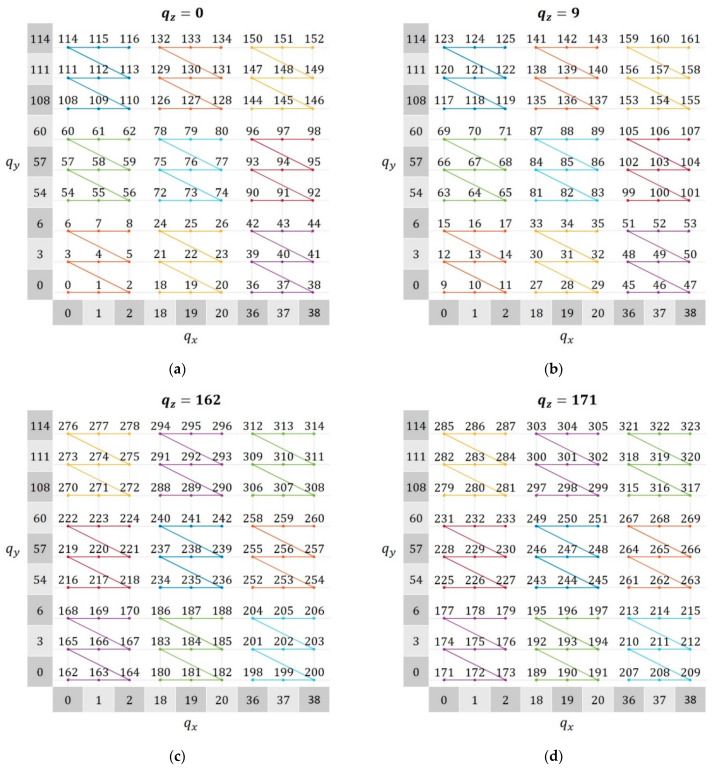
Child subimage indices c at their corresponding locations over the modified Morton order curve with the recurrent sequences and $q_{y}$ displayed on the axes and with (**a**) $q_{z}=$ 0, (**b**) $q_{z}=$ 9, (**c**) $q_{z}=$ 162, and (**d**) $q_{z}=$ 171.

**Figure 5:**
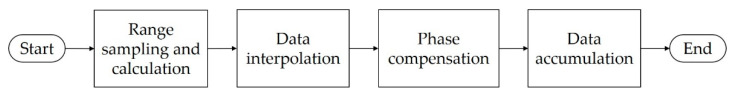
Flowchart of the process for computing child synthetic aperture radar (SAR) data.

Figure 5 demonstrates how Equations (6) and (7) correspond to the curve shown in Figure 3d–f. The sequences $q_{x}$ and $q_{y}$ are indicated on the axes, and each panel corresponds to a different element of $q_{z}$. The child subimage index starts with c=0 at the bottom left corner of Figure 4a, then moves back and forth between the layers $q_{z}=0$ and $q_{z}=9$ until reaching c=161 at the top right corner of Figure 4b. Then, it continues at the bottom left corner of Figure 4c, going back and forth between $q_{z}=162$ and $q_{z}=171$ up until the end, at the top right corner of Figure 4d.

#### 2.1.3. Computing Child SAR Data

The child SAR data are both an output of the current iteration and an input for the next. For this reason, multiple range samples are required until the second to last iteration. Additionally, the child SAR data are a function of two slant range distances instead of one. Except for these differences, computing child SAR data is the step that most resembles the direct BP algorithm. Its process is illustrated in Figure 5.

**Figure 6:**
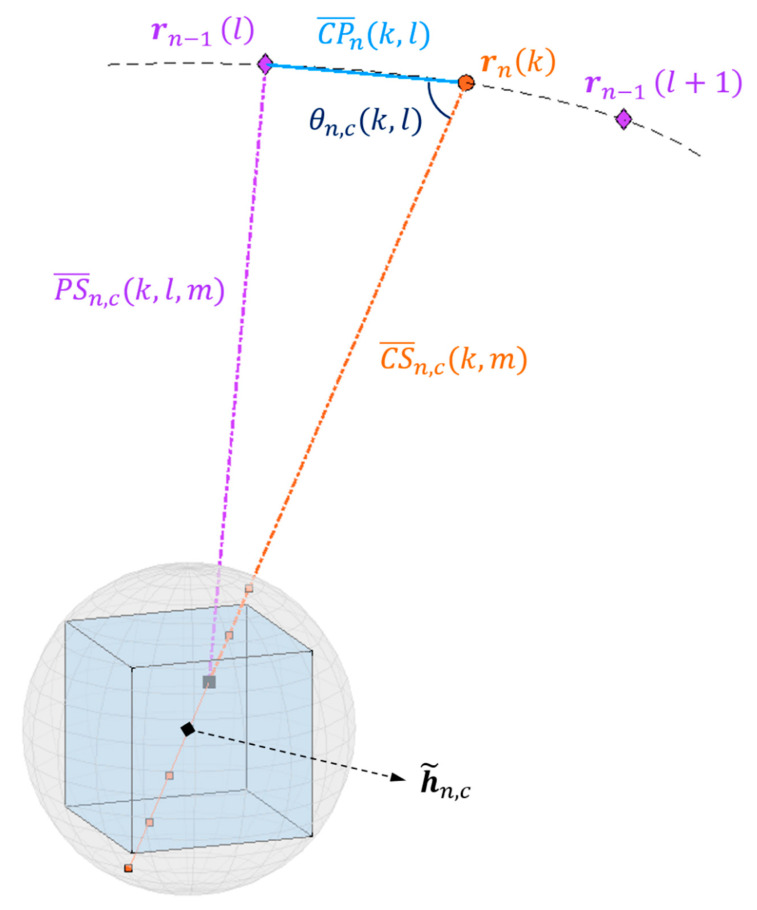
Range samples at a child subimage and geometry for calculating distances between a range sample, a child subaperture, and a parent subaperture.

Range samples are collected along a line defined by the center of the child subaperture $r_{n}\left(k\right)$ and the center of the child subimage ${\tilde{h}}_{n,c}$. A sample is always taken at ${\tilde{h}}_{n,c}$; except for the last iteration, other samples are taken along the diameter of the sphere that circumscribes the child subimage, as depicted in Figure 6. The range sampling interval is the same for all iterations. It is calculated in the preparation step, shown in Figure 1a, and is equal to the resulting range bin spacing after upsampling the root SAR data. 

Figure 6 also shows how the required slant range distances are obtained. The depicted triangle is composed of the following vertices:

- (C) the child subaperture center $r_{n}\left(k\right)$;
- (P) the parent subaperture center $r_{n-1}\left(l\right)$;
- (S) the m^th^ data sample within a child subimage centered at ${\tilde{h}}_{n,c}$.

The sides ${\bar{CP}}_{n}\left(k,l\right)$ and ${\bar{CS}}_{n,c}\left(k,m\right)$, as well as the angle $\theta_{n,c}\left(k,l\right)$ between them, are found by analytic geometry. Then, the side ${\bar{PS}}_{n,c}\left(k,l,m\right)$ is calculated with the law of cosines.

The child datum $s_{n}\left(k,m,c\right)$ is computed by the coherent sum of the parent data [11]:

(8) $$s_{n}\left(k,m,c\right)=\underset{l\in \Lambda_{n,k}}{\sum }s_{n-1}\left(l,\nu_{n,c}\left(k,l,m\right),p\right)e^{j\varphi_{n,c}\left(k,l,m\right)},$$

where $l\in \Lambda_{n,k}=\left\{kL+b|b=0,\;1,\;\ldots ,\;L-1\right\}$ is the set of parent subapertures associated with the k^th^ child subaperture. The fractional index $\nu_{n,c}\left(k,m,l\right)$ is given by [11]

(9) $$\nu_{n,c}\left(k,l,m\right)=\frac{{\bar{PS}}_{n,c}\left(k,l,m\right)-{\bar{CS}}_{n-1,p}\left(l,0\right)}{\alpha },$$

where α is the range sampling interval and ${\bar{CS}}_{n-1,p}\left(l,0\right)$ is the slant range from the parent subaperture to the first sample in the parent data. The value $s_{n-1}\left(l,\nu_{n,c}\left(k,l,m\right),p\right)$ is determined via linear interpolation, and the phase compensation term $\varphi_{n,c}\left(k,l,m\right)$ in (8) is given by [13]

(10) $$\varphi_{n,c}\left(k,l,m\right)=\frac{4\pi }{\lambda_{0}}\left[{\bar{PS}}_{n,c}\left(k,l,m\right)-{\bar{CS}}_{n,c}\left(k,m\right)\right],$$

where $\lambda_{0}$ is the radar frequency.

Each of the indices k, m, and l correspond to a different matrix dimension. Note that none of the variables denoting position (indicated in bold letters) are dependent on the data sample index m, so there is no need for a fourth matrix dimension to account for the (*x*, *y*, *z*) triplets.

After reaching the final iteration, the remaining subapertures are coherently combined. Finally, the resulting serial data are mapped into a 2D or 3D data matrix using (6) and (7).

### 2.2. The Phase Error Hypothesis

According to Ulander et al. [12], the phase error is proportional to the range error averaged over all subapertures and iterations. The range error, in turn, is introduced by the FFBP algorithm and can be estimated for each iteration. For a linear flight path, the estimated range error is proportional to the child subaperture length and the child subimage width and inversely proportional to the distance between those two entities. For nonlinear flight paths, the across-track deviation has to be taken into account. Based on this analysis, they proposed a method for keeping the phase error below a given threshold:

- Calculate the maximum subimage size for the first iteration;
- Balance the increase in subaperture length with an equivalent decrease in subimage width to keep the range error constant.

This paper investigates if the phase error standard deviation $\sigma_{\Delta \varphi }$ can be predicted by the geometric parameters at the first iteration. Moreover, instead of the subimage width, the subimage diagonal is considered as it is more relevant to the FFBP algorithm detailed in the preceding sections. Specifically, the goal is to test the following hypothesis:

(11) $$\sigma_{\Delta \varphi }\propto \beta =\frac{4\pi }{\lambda_{0}}\cdot \frac{\delta_{k}\Delta_{h}}{R_{min}},$$

where $\delta_{k}$ and $\Delta_{h}$ are the child subaperture length and the child subimage diagonal at the first FFBP iteration, respectively, and $R_{min}$ is the shortest distance from the radar to the imaged volume. 

In [12], the across-track deviation is inserted into the estimated range error equation to account for phase error degradation in nonlinear flight paths. However, the FFBP algorithm proposed in this paper does not suffer from such degradation thanks to the phase compensation term (10), as noted in [20]. That is why hypothesis (11) does not take into consideration any deviations from a linear flight path.

### 2.3. The Case Study

**Figure 7:**
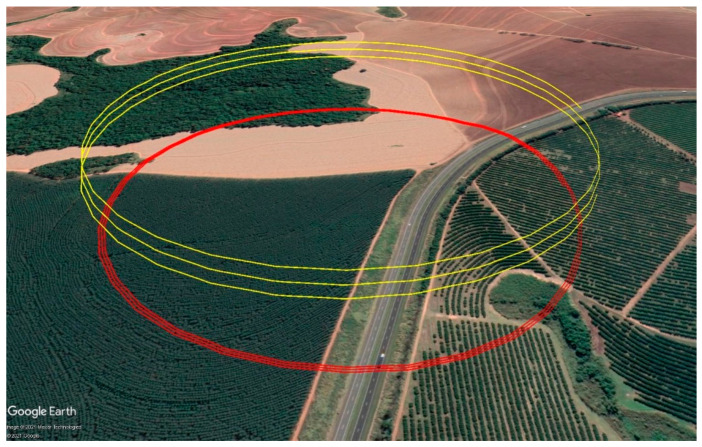
Google Earth image of the spiral flight path over the imaged area.

The case study comprised SAR data from a drone-borne SAR system [22,23] that flew over a eucalyptus plantation with a spiral flight pattern. Figure 7 displays a Google Earth image of the drone trajectory over the imaged area; the eucalyptus plantation can be seen on the bottom left. The spacing between the trees was around 3 m. The survey took place on 13 November 2019, in Mogi Guaçu, São Paulo, Brazil. The drone-borne SAR system works with three different frequency bands, but only the results for the P-band are presented here. Table 2 shows the radar acquisition parameters.

**Figure 8:**
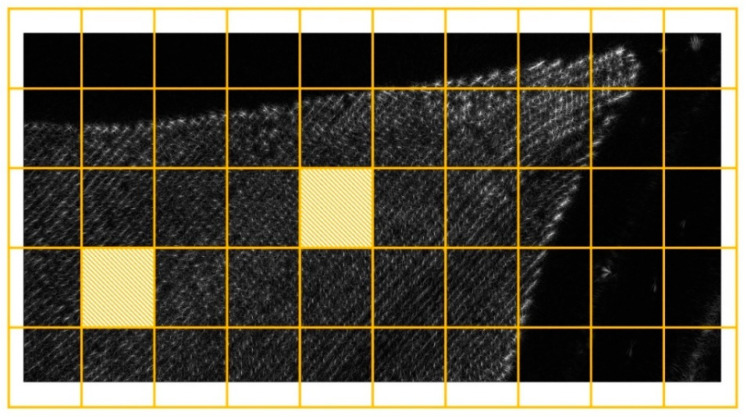
An illustration of the selected image blocks for analyzing the phase error as a function of geometric parameters.

The purpose of this case study was to investigate the hypothesis (11) by varying $\delta_{k}$, $\Delta_{h}$, and $R_{min}$ for the first iteration. This was accomplished by the following steps:

- Setting different values for the number of subapertures that are combined at each iteration (L);
- Choosing different schemes for the initial partition into image blocks;
- Selecting two image blocks for analysis, one close to the edge and one close to the center of the output image (see Figure 8).

Table 3 shows the selected set of input parameters.

For each setup, the partition scheme was the same for all image blocks. Because of that, the resulting number of pixels or voxels might not match the expected value calculated from the output image dimension and resolution. There were two options: either process the image with a different resolution or let the output image size be distinct from what is required. Because the FFBP images needed to be compared with the BP to carry out the analysis, the second option was adopted. Moreover, to minimize the waste of computing undesired pixels or voxels, the actual number of image blocks might be larger than the one provided as an input. Ultimately, this would result in a wider variation for $\Delta_{h}$ and $R_{min}$. A function executed this process at the preparation step (Figure 1a). The outcome is figuratively represented in Figure 8.

Both BP and FFBP algorithms were written in MATLAB R2018a with vectorized variables and parallel computing functions. All data were processed on an Intel(R) Core (TM) i7-7700 CPU (3.60 GHz) with 64 GB RAM.

## 3. Results

### 3.1. FFBP vs. BP

**Figure 9:**
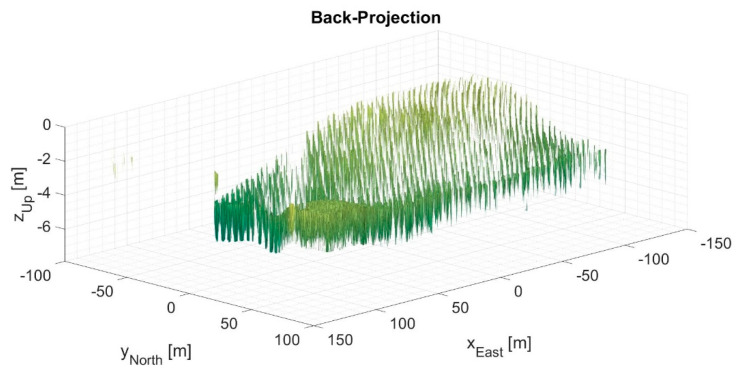
3D output image processed by the back-projection (BP) algorithm. Perspective view of isosurfaces at −15 dB normalized magnitude.

**Figure 10:**
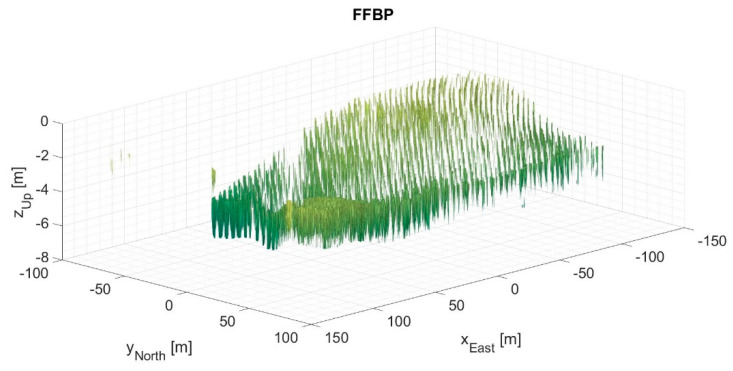
3D output image processed by the FFBP algorithm with L= 5 and an (8 × 4 × 1) initial partition. Perspective view of isosurfaces at −15 dB normalized magnitude.

Figure 9 and Figure 10 present the 3D output images processed by the direct BP algorithm and the FFBP algorithm, respectively. They depict isosurfaces at −15 dB normalized magnitude, clearly showing that the radar detects every single eucalyptus tree. The processing setup for Figure 10 uses L= 5 and an (8 × 4 × 1) initial partition. Although this setup produced the highest phase error of the case study, a qualitative comparison suggested that the differences between the two images were quite subtle. Indeed, the degree of coherence between them was 0.9916; the magnitude error had a −0.3 dB mean and a 2.5 dB standard deviation; the mean phase error was 0.0007 rad (0.04°); and the phase error standard deviation was 0.35 rad (19.9°), slightly below π/8 rad.

**Figure 11:**
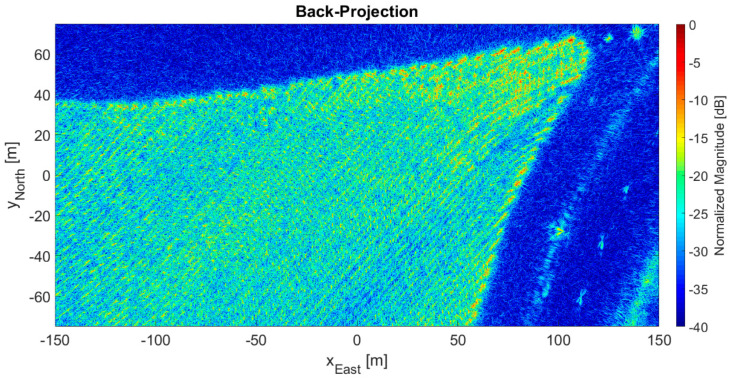
2D output image processed by the BP algorithm.

**Figure 12:**
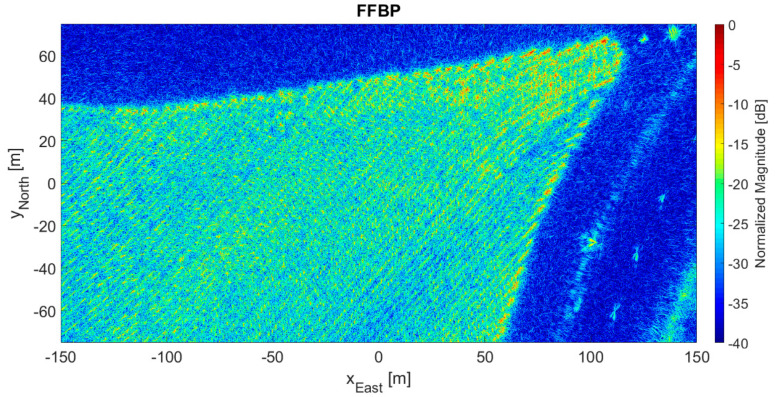
2D output image processed by the FFBP algorithm with L= 5 and an (8 × 4 × 1) initial partition.

Figure 11 presents the 2D output image processed by the direct BP algorithm, and Figure 12 shows the 2D output image processed by the FFBP algorithm using L= 5 and an (8 × 4 × 1) initial partition. Again, this setup produced the highest phase error of the case study. However, as in the previous case, the differences between the two images were barely perceptible. The degree of coherence between them was 0.9942; the magnitude error had a −0.2 dB mean and a 2.3 dB standard deviation; the mean phase error was 0.0004 rad (0.02°); and the phase error standard deviation was 0.33 rad (18.8°), also somewhat below π/8 rad. Lastly, the lines of trees of the eucalyptus plantation can be easily seen in both Figure 11 and Figure 12.

### 3.2. Phase Error vs. SNR

**Figure 13:**
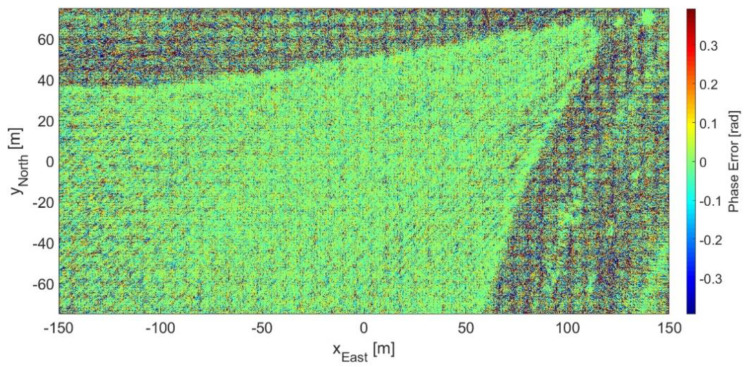
Phase error for the 2D FFBP image with L= 5 and an (8 × 4 × 1) initial partition.

Figure 13 presents the phase error response between the 2D images shown in Figure 11 and Figure 12. Notice that the darkest area of Figure 11 corresponds to an increase of phase error in Figure 13, which indicates a noisy behavior. The mean normalized magnitude at a 30 × 30 m^2^ square in the northwestern-most corner of Figure 11 is close to −40 dB. Thus, this value was considered the noise floor level for calculating the SNR for the following analysis.

**Figure 14:**
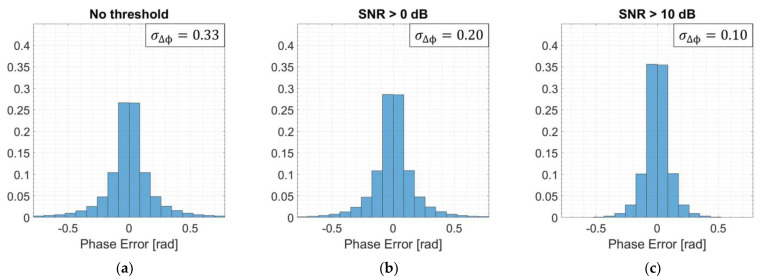
Histogram of the phase error for the 2D FFBP image with L= 5 and an (8 × 4 × 1) initial partition for (**a**) no signal-to-noise ratio (SNR) threshold, (**b**) SNR >0 dB, and (**c**) SNR >10 dB.

Figure 14 displays three histograms for the phase error. The first (Figure 14a) had no SNR threshold, i.e., all pixels are taken into account; the second (Figure 14b) had a 0 dB SNR threshold; and the last one (Figure 14c) had a 10 dB SNR threshold. As can be seen, between Figure 14a,b, there is a subtle change of less than 2% in relative probability for each bin. The corresponding decrease in phase error standard deviation was from 0.33 (18.8°) to 0.20 (11.4°) rad. On the other hand, between Figure 14a,c, there is a perceptible change of more than 8% in relative probability for the central bins, which made the phase error standard deviation decrease even more to 0.10 rad (5.8°). 

As the 10 dB SNR threshold might have eliminated valuable information, the chosen threshold for the subsequent analysis was 0 dB SNR. By applying the selected SNR threshold to Figure 10, the resulting phase error standard deviation became 0.22 rad (12.7°).

### 3.3. Phase Error vs. Geometric Parameters

**Figure 15:**
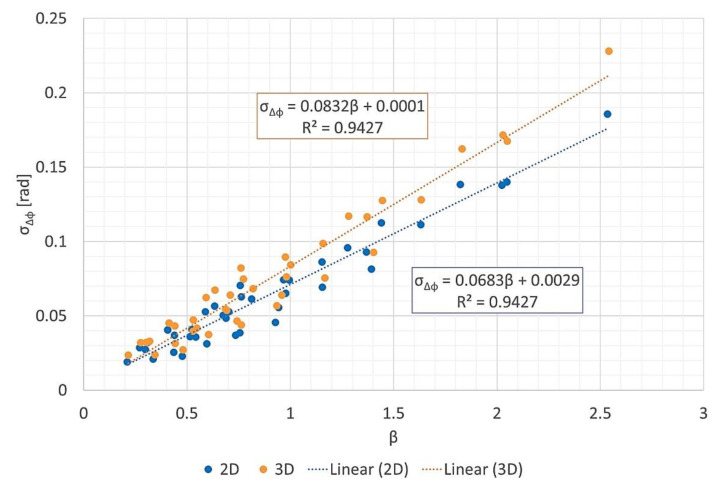
Phase error standard deviation $\sigma_{\Delta \phi }$ vs. β, with separate linear regressions for 2D and 3D data.

**Figure 16:**
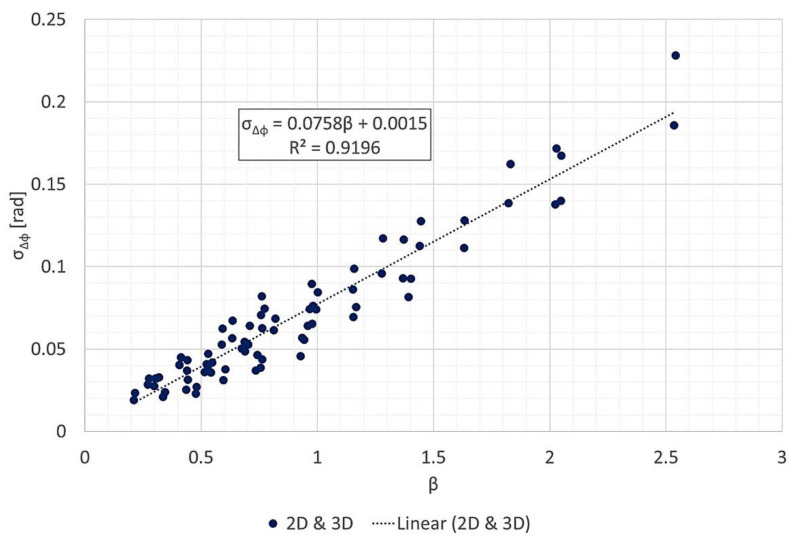
Phase error standard deviation $\sigma_{\Delta \phi }$ vs. β, with the same linear regression for both 2D and 3D data.

Figure 15 and Figure 16 present scatter plots of the phase error standard deviation $\sigma_{\Delta \phi }$ versus β, defined by (11). Figure 15 shows two separate linear regressions for 2D and 3D images, while Figure 16 shows a unique linear regression for all results. In Figure 15, the slopes indicate lower phase errors for the 2D data than for the 3D data.

The statistics for all three linear regression models are indicated in Table 4. All intercepts had high *p*-values, and all of their confidence intervals contained zero. Thus, the intercepts were not statistically significant. On the other hand, all slopes had negligible *p*-values, and neither of their confidence intervals contained zero. Moreover, all linear regression models presented high coefficients of determination, $R^{2}>0.9$. Therefore, the hypothesis $\left(\sigma_{\Delta \phi }\propto \beta \right)$ is supported by the data. Moreover, the hypothesis is accepted even when combining 2D and 3D data (Figure 16).

### 3.4. Phase Error vs. Time

**Figure 17:**
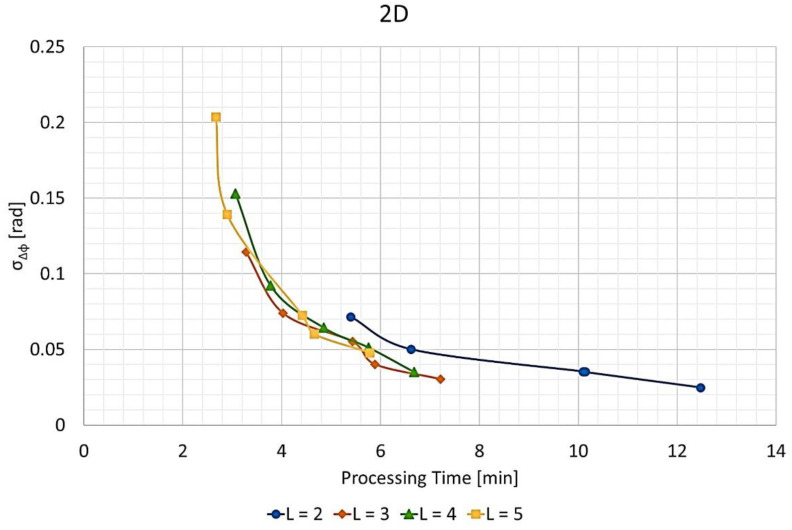
Phase error standard deviation $\sigma_{\Delta \phi }$ vs. processing time for the 2D output images and different values of L.

**Figure 18:**
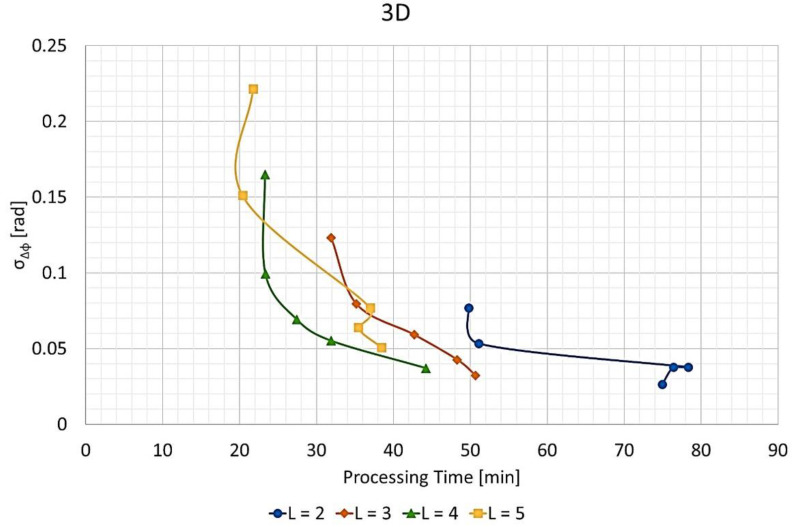
Phase error standard deviation $\sigma_{\Delta \phi }$ vs. processing time for the 3D output images and different values of L.

Figure 17 presents the phase error standard deviation versus the processing time for the 2D images at different values of L. Figure 18 shows a similar line chart for the 3D images. Here, the phase error standard deviation was calculated for the whole image, not only for the selected image blocks of Figure 8. 

As can be seen, the curves for the 3D images (Figure 18) are not as smooth as those for the 2D images (Figure 17). The reason is that the function for defining the split scheme causes unnecessary waste and needs improvement. Beyond that, it is easy to notice that both curves for L= 2 are far slower than for other values of L.

Table 5 lists the slowest, fastest, and average processing times of the FFBP algorithm compared to the BP for 2D and 3D images. Table 5 also presents the corresponding speed-up factors. These results are from the same data sets of Figure 17 and Figure 18. It is worth noticing that the speed-up factor was more pronounced for the 3D images.

## 4. Discussion

The hypothesis that the geometric parameters at the first iteration can predict the phase error standard deviation at the output was validated for the P-band data. It was also validated when joining the 2D and 3D data sets (Figure 16), reinforcing the idea that what matters most for this FFBP algorithm is the diagonal of the subimages, not their width. In Section 3.3, all linear regression models produced slopes with negligible *p*-values, statistically irrelevant intercepts, and $R^{2}>$ 0.9.

This hypothesis was inspired by the range error analysis presented in [12] but disregarding the effect of any deviations from a linear flight path. The reason is that the phase compensation term (10) ensures good focusing quality for nonlinear flight patterns. This term was proposed by Zhang et al. [13] but with a different goal, namely to avoid taking range samples at each recursion in order to accelerate processing.

**Figure 19:**
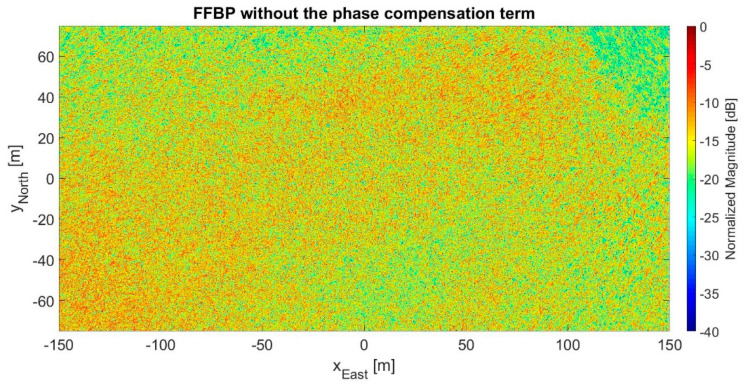
2D output image processed by the FFBP algorithm without the phase compensation term (10) for the setup with L= 2 and a (24 × 12 × 1) initial partition.

If (10) was removed from the FFBP algorithm, the outcome of the case study presented here would be completely unsatisfactory. Indeed, Figure 19 shows the resultant 2D image with L= 2 and a (24 × 12 × 1) initial partition, i.e., the configuration with the lowest phase error standard deviation in Section 3.4. If Figure 19 is compared to the BP output image of Figure 11, the degree of coherence is a meager 0.12.

According to the method for controlling the phase error proposed in [12] (and briefly described in Section 2.2), the partition scheme should attempt to keep the product of the subimage diagonal by the subaperture length constant across all iterations. This was possible in the processing of the 2D images but not for the 3D images. The reason is that the number of voxels in the *x*- and *y*-directions are significantly larger than in the *z*-direction. Therefore, in some setups, the volumetric images were only split across the *x*- and *y*-directions for the last iterations. Consequently, the linear regression of the 3D image data set had a slightly steeper slope than that of the 2D data set.

In the future, this methodology should be repeated for other frequencies as the phase error also depends on the radar wavenumber. The linear regression models can be used to determine processing parameters from a requirement in phase error, which would be more accessible for other users to benefit from the FFBP algorithm.

As expected, the configuration with the lowest image quality (see Figure 10 and Figure 12) had the longest subaperture length and subimage diagonal, i.e., L= 5 with an (8 × 4 × 1) initial partition. Likewise, the configuration with the highest image quality had the shortest subaperture length and subimage diagonal, i.e., L= 2 with a (24 × 12 × 1) initial partition. Table 6 lists some figures of merit at these extremes for the 2D and 3D data sets, namely the phase error standard deviation, the degree of coherence, and an SNR of equivalent thermal noise, calculated according to [26]. SNR of equivalent thermal noise can be understood as the signal-to-thermal noise ratio that would result in an interferometric image with the same degree of coherence. Table 6 also shows the values for an average image quality, which corresponds to the following configurations:

- L= 5 with a (16 × 8 × 1) initial partition for 2D;
- L= 2 with an (8 × 4 × 1) initial partition for 3D.

It is important to note that the term “lowest quality” refers to a relative comparison within the data set, not to poor quality in absolute terms. Qualitatively, Figure 10 and Figure 12 appear to be almost identical to Figure 9 and Figure 11, which may well indicate that this level of image quality is suitable for SAR processing. Indeed, in [23], the same drone-borne SAR system produced a high-accuracy forest inventory with SAR interferometry in the P-band. A 5% accuracy was possible thanks to the forest SNR being higher than 17 dB. Because the SNR of equivalent noise was more than 20 dB, the configurations with the lowest image quality were already satisfactory. Moreover, they were also associated with the fastest processing times (see Table 5), with speed-up factors of 13 and 21 for 2D and 3D images, respectively.

On the other hand, the configurations with the highest image quality had unnecessarily slow processing times. If a specific application would require an SNR higher than 20 dB, then a configuration with average image quality could be employed. The average phase error standard deviation points were close to those with average processing time in Figure 17 and Figure 18. Therefore, more demanding applications could benefit from a speed-up factor of about 6 for 2D images and 10 for 3D images.

Figure 9, Figure 10, Figure 11 and Figure 12 show processed images from data acquired with a spiral flight path. As can be seen, the trees on the eucalyptus plantation are easily recognized. If the same area was surveyed with a linear flight pattern, the resultant image would have a slant range resolution of 3 m and an azimuth resolution of 50 cm [23]. However, thanks to the 360° acquisition, the resolution across all directions in the (*x*, *y*) plane was at least 50 cm. The maximum attainable resolution in the (*x*, *y*) plane would be $∼\lambda_{0}/4$ [1,2,3].

Unsurprisingly, the speed-up factor was higher for 3D images than for 2D images. Section 2.1 pointed out that FFBP algorithms can reduce the computational cost from $O\left(N^{3}\right)$ to $O\left(N^{2}\log N\right)$. Therefore, the expected speed-up factor N/logN would increase with the size of the output image. 

It was noted in Section 3.4 that the function for creating the partition scheme needs improvement. Moreover, the current version of the algorithm assumes that the radar is constantly illuminating the imaged area. In future works, this assumption should no longer be required. Finally, a bistatic version of the algorithm should be implemented as well.

## 5. Conclusions

Spiral and multicircular SAR acquisition techniques can produce high-resolution 3D SAR images. In [20], the authors presented an FFBP algorithm capable of processing simulated SAR data replicating a spiral flight path. In the present work, an improved version of the FFBP algorithm [21] could successfully process real P-band SAR data acquired by a drone-borne SAR system that performed a spiral flight pattern.

This paper proposes a statistical phase error analysis to determine how the FFBP setup affects the quality of the output images. In the case study, the same raw radar data were processed with the FFBP algorithm with different parameters to produce several 2D and 3D SAR images. The analysis validates the hypothesis that geometric parameters defined at the beginning of processing can predict the phase error standard deviation at the output. In future works, the linear regression models generated in the analysis could be applied to determine the processing setup from a requirement in phase error.

The FFBP algorithm produces nearly identical images to those processed with a direct BP algorithm, only faster. The speed-up factor is up to 21 times for the 3D images and 13 times for 2D images, with a phase error standard deviation of ~12°, corresponding to an SNR of equivalent thermal noise of 20 dB. For higher image quality, with a phase error standard deviation of ~4° and 30 dB SNR of equivalent thermal noise, the speed-up factor is 10 and 6 times for the 3D and 2D images, respectively.

## Figures and Tables

**Table 1:** Order of arrangement of the *x*, *y*, and *z* coordinates of the child subimage centers in a modified Morton order curve with a (3 × 3 × 2) partition.

d	$\tilde{x}\left(d\right)$	$\tilde{y}\left(d\right)$	$\tilde{z}\left(d\right)$	d	$\tilde{x}\left(d\right)$	$\tilde{y}\left(d\right)$	$\tilde{z}\left(d\right)$
0	x(0)	y(0)	z(0)	9	x(0)	y(0)	z(1)
1	x(1)	y(0)	z(0)	10	x(1)	y(0)	z(1)
2	x(2)	y(0)	z(0)	11	x(2)	y(0)	z(1)
3	x(0)	y(1)	z(0)	12	x(0)	y(1)	z(1)
4	x(1)	y(1)	z(0)	13	x(1)	y(1)	z(1)
5	x(2)	y(1)	z(0)	14	x(2)	y(1)	z(1)
6	x(0)	y(2)	z(0)	15	x(0)	y(2)	z(1)
7	x(1)	y(2)	z(0)	16	x(1)	y(2)	z(1)
8	x(2)	y(2)	z(0)	17	x(2)	y(2)	z(1)

**Table 2:** Radar acquisition parameters.

Radar Parameters	Values	Units
Carrier wavelength	70.54	cm
Bandwidth	50	MHz
Range resolution	2.4	m
Pulse repetition frequency	64.95	Hz
Mean velocity	8.5	m/s
Mean flight radius	338	m
Height above ground level	79–120	m
Number of turns	3	-

**Table 3:** Set of processing parameters for the case study.

Processing Parameter		Values
Output image dimension	2D	300 × 150 m^2^
	3D	300 × 150 × 2.4 m^3^
Output image resolution	2D	0.2 × 0.2 m^2^
	3D	0.2 × 0.2 × 0.2 m^3^
Number of subapertures combined at each iteration (L)		2
		3
		4
		5
Initial partition of image blocks		8 × 4 × 1
		12 × 6 × 1
		16 × 8 × 1
		20 × 10 × 1
		24 × 12 × 1

**Table 4:** Statistics of the linear regression models.

Data	Coefficient	Estimate	Standard Error	95% Confidence Interval		*p*-Value	$R^{2}$
2D	Intercept	0.0029	0.0028	−0.0029	0.0086	0.32	0.9427
	Slope	0.0683	0.0027	0.0628	0.0738	3.3 × $10^{-25}$	
3D	Intercept	0.0001	0.0035	−0.0069	0.0071	0.97	0.9427
	Slope	0.0832	0.0033	0.0765	0.0899	3.3 × $10^{-25}$	
2D and 3D	Intercept	0.0015	0.0026	−0.0038	0.0067	0.58	0.9196
	Slope	0.0758	0.0025	0.0708	0.0809	1.9 × $10^{-44}$	

**Table 5:** Processing time of the slowest, fastest, and average FFBP configurations compared to the BP algorithm.

Image Type	BP Processing Time	FFBP		
		Configuration	Processing Time	Speed-Up Factor
2D		Fastest	2 min 40 s	13.33
	35 min 33 s	Average	5 min 45 s	6.18
		Slowest	12 min 28 s	2.85
3D		Fastest	20 min 24 s	21.2
	7 h 12 min 18 s	Average	42 min 8 s	10.3
		Slowest	1 h 18 min 18 s	5.52

**Table 6:** Performance of the configurations with highest, average, and lowest image quality.

Image Type	Figure of Merit	Image Quality		
		Highest	Average	Lowest
2D	Phase Error Standard deviation	0.025 rad (1.4°)	0.073 rad (4.2°)	0.20 (11.7°)
	Degree of coherence	0.9999	0.9993	0.9945
	SNR of equivalent Thermal noise	40 dB	31 dB	23 dB
3D	Phase error Standard deviation	0.026 (1.5°)	0.077 rad (4.4°)	0.22 rad (12.7°)
	Degree of coherence	0.9999	0.9988	0.9921
	SNR of equivalent Thermal noise	38 dB	29 dB	21 dB

## Data Availability

The data presented in this study are openly available in Zenodo at doi:10.5281/zenodo.4883258, reference number [21].
